# Early loss of radiographic reduction after acute acromioclavicular joint reconstruction: Comparison of open Double Endobutton fixation vs. Nottingham Surgilig

**DOI:** 10.1051/sicotj/2022044

**Published:** 2022-11-25

**Authors:** Georgios Saraglis, Harsh Chaudhari, Shahrukh Reza Sanjani, Anwar Khan

**Affiliations:** Department of Trauma and Orthopaedics, Luton and Dunstable University Hospital, Bedfordshire NHS Trust Luton LU4 0DZ UK

**Keywords:** Acromioclavicular joint dislocation, ACJ dislocation, Double Endobutton, Nottingham Surgilig, Tightrope system, LockDown system

## Abstract

*Introduction*: Surgical treatment is usually recommended for the acute unstable acromioclavicular joint (ACJ) dislocations. Among the wide variety of different surgical techniques, the Double Endobutton and the Nottingham Surgilig technique are two of the most widely acceptable and well described techniques. The aim of this study was to offer a direct comparison of the above techniques in question, analysing the patients outcomes and assessing the risk of early loss of radiographic reduction. *Materials and methods*: A total of 48 patients who met the inclusion criteria were included in the study. Patients were categorised in two groups (Endobutton and Nottingham Surgilig group) and post operative assessment of the patients was performed using the Oxford Shoulder (OSS) and Constant Murley (CMS) scores. Patient demographics, hand dominance, ACJ classification and co-morbidities were included in the analysis and radiographic evaluation was conducted for both groups. *Results*: Both techniques provide a good outcome in the management of unstable ACJ dislocations but the risk of early radiographic failure remains higher in the double Endobutton technique (26% vs. 17.39% for the Nottingham Surgilig group). Factors such as patients’ demographics, hand dominance, co-morbidities and grade of ACJ separation do not seem to contribute to radiographic loss of reduction, whereas the incorrect positioning of the coracoid endobutton is a significant factor predisposing to early radiographic failure, *P* < 0.001. *Discussion*: The incidence of early loss of radiographic reduction still remains high in both groups. In order to reduce this common complication, accurate placement of the coracoid endobutton under fluoroscopic intra-operative control is strongly recommended.

## Introduction

Acromioclavicular joint (ACJ) dislocations are considered common shoulder injuries (9% of all shoulder girdle injuries) [1], with the Rockwood classification (I–VI) being the most commonly used based on the radiographic description of the ACJ and the congruity of the coracoclavicular ligaments (CC) [2].Types I–II are usually treated conservatively, whereas surgical intervention is indicated for type IV–VI due to the disruption of the CC ligaments [3, 4].

Several surgical techniques have been described to reconstruct the CC ligaments, however, the optimal reconstructive technique has not been described [5, 6]. Non-anatomical CC ligament reconstruction techniques are becoming less popular, with modern anatomical CC reconstruction techniques focusing on both vertical and horizontal plane stability gaining popularity in the recent literature [7]. However, as new techniques have been introduced more complications have been described with the most well-described failure being the early loss of radiological reduction [7, 8].

The purpose of this study was to compare the results of two of the most well-described techniques used for the management of acute unstable ACJ dislocations: Double Endobutton (AC Tightrope, Arthex, Naples) and Nottingham Surgilig implant. Secondary objectives included analysing the influence of the post-operative results were patients’ demographics, type of ACJ dislocation, comorbidities and hand dominance. The hypothesis of our study was that the radiographic failure rate would be equal in both groups (Endobutton and Nottingham Surgilig group).

## Materials and methods

Approval was obtained from the Institutional Board of our institution and all patients included in the above study formally consented. In this retrospective review of acute ACJ dislocations, patients were identified by searching our institution’s electronic database using the procedure codes linked to the above surgical techniques in question. Inclusion criteria were patients with an isolated ACJ injury (Rockwood IV and above) with a minimum follow-up of 2 years, patients who underwent surgical procedure within 3 weeks from the day of injury (acute injuries), patients who underwent a procedure with the Nottingham Surgilig or Endobutton system. Patients with associated fracture or polytrauma, patients who underwent hook plate fixation or other surgical techniques and patients that underwent their procedure later than 3 weeks from the date of injury were excluded from our study. Rockwood classification type 3 injuries, were also excluded from the study, as in our institution Grade 3 injuries are initially treated conservatively and surgical treatment is offered when conservative treatment fails (not included in the acute, within 3 weeks timeframe, period of this study).

Patient demographics, hand dominance, comorbidities, and ACJ dislocation grade were assessed for all the patients included in the study (Table 1). Post-operative anteroposterior radiographs were reviewed by all the authors to determine the loss of radiographic reduction. For all the patients included in the study, a comparison was made of the immediate post-operative radiograph with the radiograph at a 3 month interval and the increase of the CC distance was noted for all the patients. Radiographic failure was defined as an increased CC interval of >5 mm compared with the immediate post-operative radiographs, as indicated in similar studies in the literature [9].

**Table 1:** Univariate predictors for radiographic failure.

	Tightrope system group	Nottingham Surgilig	*P*-value
Age	34.8 ± 9.1	36 ± 7.4	*P* > 0.6
Gender	Male/female: 19/4	Male/female: 22/3	*P* > 0.1
Dominant hand	21.7%	20%	*P* > 0.5
Grade of ACJ dislocation (Grade 4 and 5)	Grade 4: 69.5% (16 cases)	Grade 4: 76% (19 cases)	*P* > 0.5 (Fisher’s exact test)
	Grade 5: 30.5% (7 cases)	Grade 5: 24% (6 cases)	

## Statistical analysis

During the statistical analysis, we compared the post-operative Oxford Shoulder Score (OSS) and Constant Murley Score (CMS) at a 6-month follow-up appointment. The comparison between the two groups was performed using the unpaired *t*-test. Continued variables with parametric distribution were presented as means and standard deviations whereas non-parametric distributions were as medians and percentiles. For categorical variables, Fisher’s exact test was used (Table 2).

**Table 2:** Radiographic failure by surgical technique.

Technique used	Number of cases	Missing radiographs at 3 months review	Number of radiographic failures (increase in the CC distance > 5 mm)	Failure (%)
Double Endobutton	23	–	6	26%
Nottingham Surgilig	25	2	4	17.39%
				*P* = 0.01

## Results

A total of 48 cases performed by 2 surgeons for isolated acute ACJ dislocation were identified through our database search. The mean age of the Tightrope system group (23 patients) was mean 34.8 with a standard deviation (*SD*) of 9.1 (mean = 34.8 ± 9.1). The mean age of the Nottingham Surgilig group (25 patients) was 36 ± 7.4, *P* > 0.6.

The comparison of the CMS and for both techniques did not reveal any statistically significant difference between the two groups of the study: the mean CMS for the Tightrope system group at 6 months follow-up assessment showed a mean of 83 ± 4.6, whereas for the Nottingham Surgilig group 82.4 ± 3.9, 0.1 < *P* < 0.5.

The OSS assessment at 6 months revealed similar satisfactory results in both groups, Tightrope system mean 15.43 ± 1.7 and Nottingham Surgilig mean 14.6 ± 2.1, *P* > 0.1.

Factors such as age, gender, hand dominance and the grade of ACJ separation do not seem to predispose to a higher rate of early radiographic failure. The statistical analysis of the patient’s demographics and the Rockwood grade of ACJ dislocation (Table 1) did not seem to be statistically related to the early onset of radiographic failure, with similar findings reported by other authors [12, 13].

## Radiographic failure

All post-operative radiographs were reviewed and 2 patients of the Nottingham Surgilig group were excluded for lack of post-operative imaging at the 3-month follow-up appointment. In total, 23 patients of the Tightrope and 23 patients of the Nottingham Surgilig group were included in the radiological assessment (7 female, 39 male) with radiographic follow-up (mean = 4.2 months). The radiographic appearance of the CC distance was measured in all patients on anterioposterior radiographs of both clavicles, according to the same practice indicated by other authors [10].

Both techniques seem to provide an adequate reduction of the CC distance during the immediate post-operative period, however, a higher rate of early loss of radiographic reduction was noted in the Endobutton group.

For the Endobutton group (23 patients) the mean CC distance during the immediate post-operative radiograph was 12.64 ± 2.9 and for the Nottingham Surgilig group (25 patients) 11.43 ± 3.2, *P* > 0.1.

**Graph 1:**
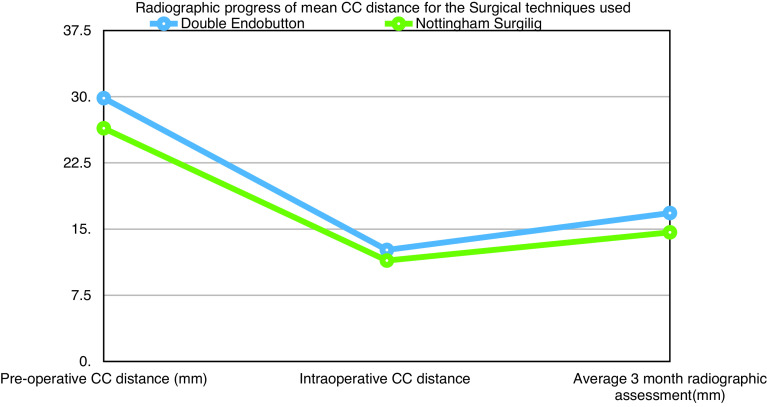
Radiographic progress of mean CC distance in the two techniques.

During the radiographic follow-up, the mean CC distance of the Tightrope group (23 patients) increased to 16.82 ± 3.1 and for the Nottingham Surgilig group (23 patients) to 14.62 ± 2.9, *P* = 0.01 (Graph 1).

**Figure 1:**
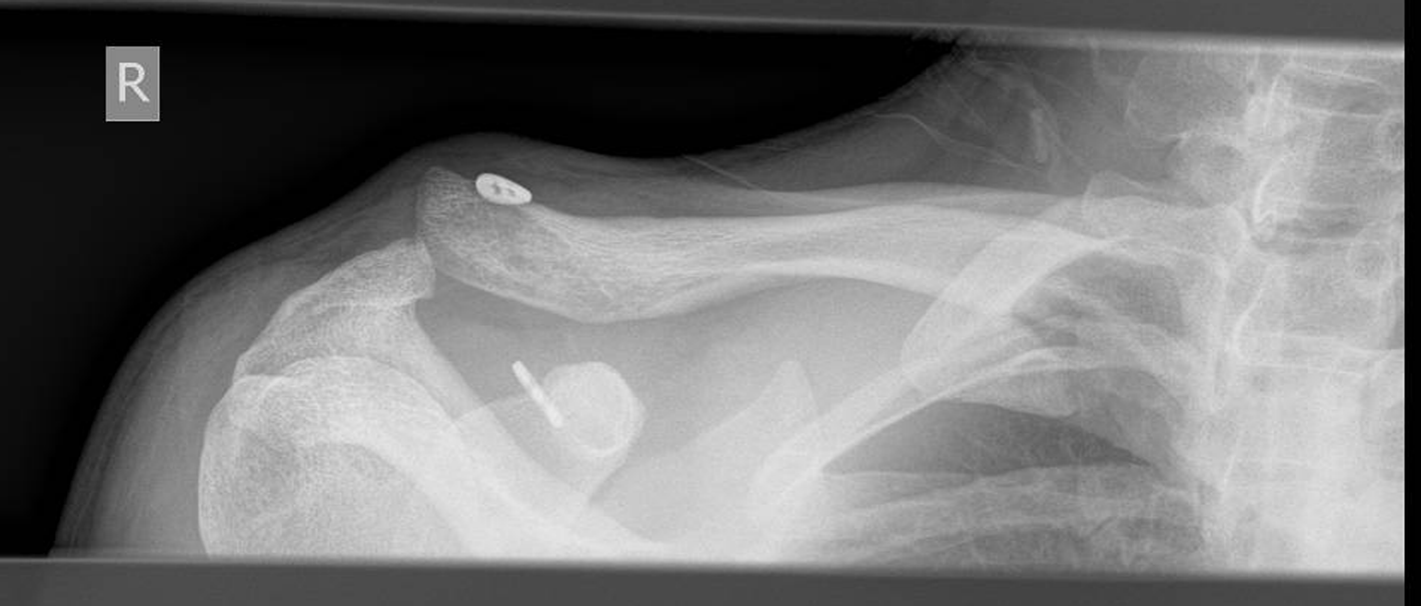
Example of lateral subluxation of the coracoid endobutton leading to loss of radiographic reduction.

**Figure 2:**
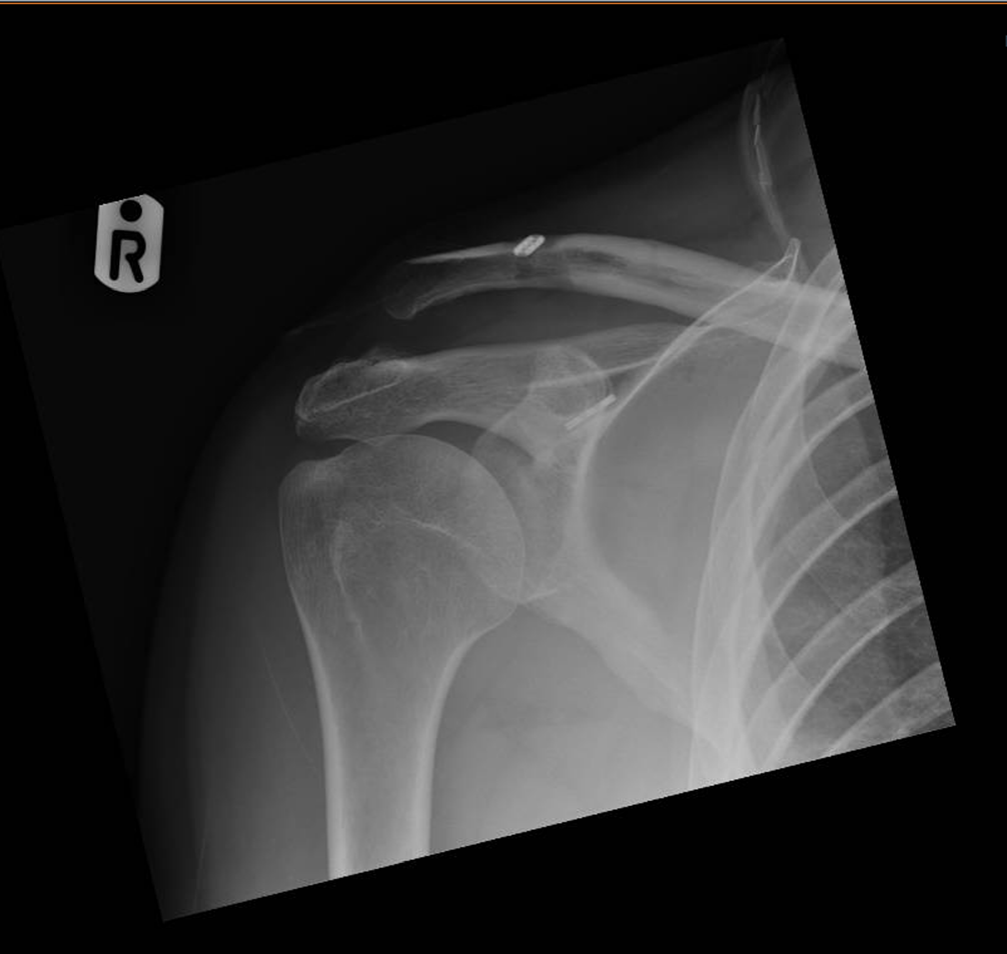
Example of medial mal-positioning of the coracoid tunnel, leading to loss of CC reduction and post-operative subluxation of the coracoid endobutton.

**Figure 3:**
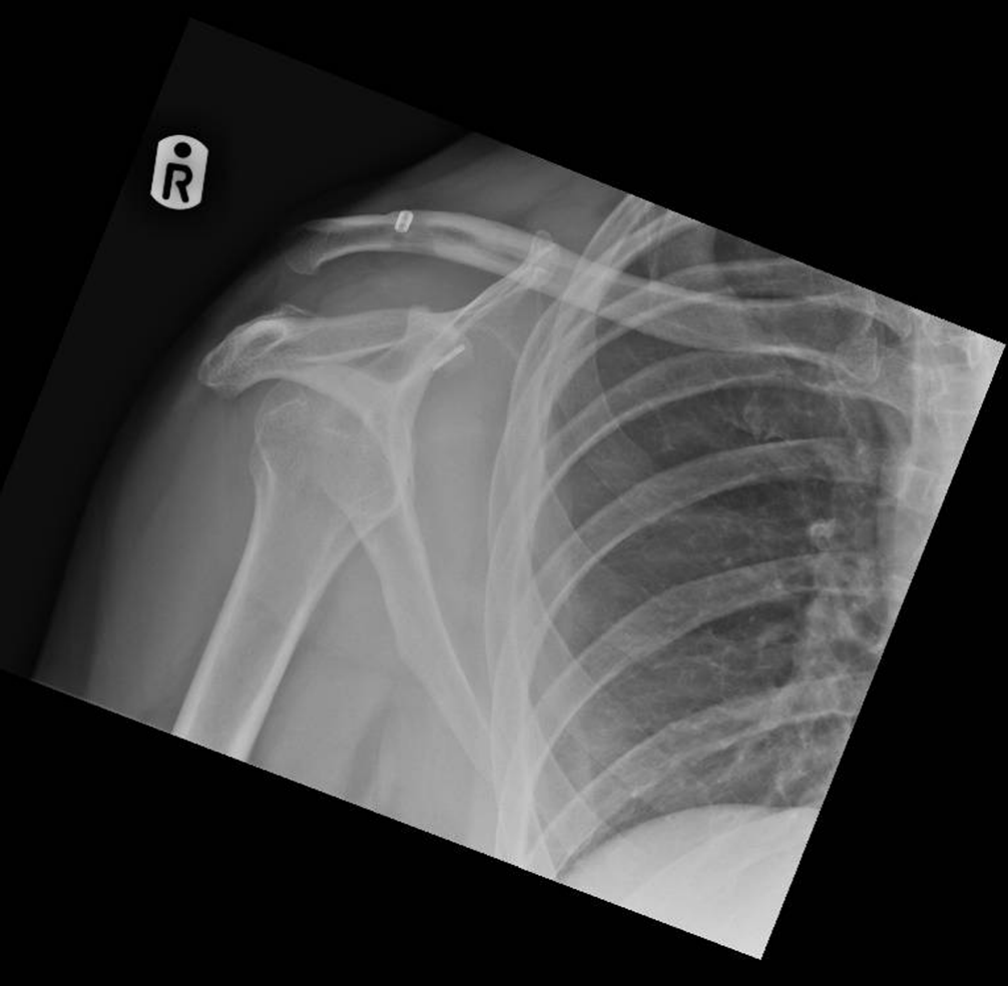
Medial subluxation of the coracoid button, 3 months post-operatively due to medial coracoid tunnel positioning.

A subsequent review of the cases presenting with radiological failure of both techniques led us to separately evaluate possible factors predisposing to post-operative failure. Factors analysed were: the radiological positioning of the coracoid endobutton (for the Endobutton group), evidence of osteolysis on the 3 months radiograph and the presence of ectopic calcification. In total, 10 cases were included in the subsequent radiographic evaluation (6 cases from the Endobutton and 4 cases from the Nottingham Surgilig group) Table 3.

**Table 3:** Factors assessed during the subsequent evaluation of radiographic failure cases.

	No of radiological failure cases	Osteolysis	Ectopic calcification	Coracoid endobutton
Double Endobutton	6	1 case	No cases	2 cases of lateral placement, 1 case of medial placement, 50%
Nottingham Surgilig	4	1 case	1 case	–
*P*-value		*P* > 0.1	*P* > 0.1	*P* < 0.001

There was one case of osteolysis noted in each group, during the 3 months post-operative radiograph review and one case of early ectopic calcification in the Nottingham Surgilig group, but no statistical significance was noted among the two groups, *P* > 0.1. One of the most interesting radiographic findings of this evaluation was the significance of the correct positioning of the coracoid endobutton (Figs. 1–3).

## Complications

In the Nottingham Surgilig group, complications were observed in 3 patients, 2 cases of superficial skin infection, treated conservatively with the course of oral antibiotics for 7 days and 1 case of deep surgical site infection which required complete removal of the implant with no further surgical intervention. In the Endobutton group, one case of implant failure was noted, which required revision ACJ reconstruction using a different implant. Complications mentioned in other studies [11] such as subacromial impingement, coracoid fracture or post-operative clavicle fracture were not noted in this study.

## Discussion

Despite recent advances in the management of ACJ dislocations [7], the Double Endobutton and Nottingham Surgilig still remain among the most widely available surgical techniques used. The present study suggests that the Nottingham Surgilig implant for the management of acute unstable ACJ dislocations was associated with improved radiographic survival in our patients, with reduced loss of the radiographic CC distance at the 3-month interval in comparison to the Tightrope system group (Graph 1). As indicated by the OSS and CM scores, both patient groups improved significantly following the surgical intervention but the statistical analysis failed to detect any statistically significant difference between the two surgical techniques (*P*-value OSS and CMS > 0.1).

The radiological assessment of the surgical techniques though, revealed that in both techniques a statistically significant loss of radiographic reduction of the CC distance was noted at the 3-month follow-up appointment (Table 2, *P* = 0.01).

Among the limitations of this study were its small sample size (48 patients), relatively short follow-up period and the presence of two different operating surgeons (Endobutton vs. Nottingham Surgilig group). Another limitation is the absence of arthroscopic Endobutton fixation techniques, which could be linked to a reduced radiographic failure rate. To our knowledge though, this is the first study including a direct comparison of the Endobutton technique vs. the Nottingham Surgilig implant for the management of ACJ dislocations, focusing on the early loss of radiographic reduction following the above techniques.

Multiple factors have been introduced in the literature associated with loss of radiographic reduction for ACJ dislocation including surgical technique, implant failure, patient factors and weight-bearing time from surgery [12]. In our study (Table 1) factors such as age, gender, Rockwood classification and hand dominance had no statistically significant correlation with the loss of radiographic reduction with similar results supported by other studies in the recent literature [13].

The correct positioning of the coracoid tunnel has been described as a prognostic factor of radiographic failure [14,15], with medial and lateral dislocations/subluxations of the coracoid button being well described in the literature increasing the probability of further loss of radiological reduction. In the radiographic failure group of our study, 50% of the Double Endobutton group had the coracoid button placed either medially or laterally to the centre of the coracoid (2 cases laterally and 1 case medially), *P* < 0.001. As indicated by other authors [14], the correct positioning and drilling of the coracoid tunnel can significantly contribute to radiological failure. Ferreira et al. [14] demonstrated that center-to-center coracoid tunnel drilling seems to offer a reduced risk of post-operative reduction loss.

In our study, all cases of double Endobutton fixation followed the same surgical standards (same operating surgeon): the coracoid tunnel is drilled first at the centre of the base of the coracoid process under continuous fluoroscopic guidance, followed by the drilling tunnel of the clavicle and the deployment of the Endobutton in the undersurface of the coracoid is confirmed intra-operatively via fluoroscopy.

For the Nottingham Surgilig group, meaningful radiographic assessment is difficult to be performed due to the nature of the braided synthetic polyester ligament used. The technique involves looping the ligament around the coracoid and securing it to the distal clavicle using a screw, providing secure fixation. Equally, cases of misplacement or incorrect positioning of the polyester loop around the coracoid have been described in the literature [16], but are difficult to be evaluated and confirmed radiographically on the post-operative radiographs.

## Conclusions

From our retrospective study and statistical analysis, both the Nottingham Surgilig and the Double Endobutton device provide similar outcomes in the management of ACJ dislocations at a 2-year follow-up. The early loss of radiographic reduction still remains a common pitfall for both techniques, with the correct radiographic positioning of the coracoid button (for the Endobutton group) being the most well-described. While both techniques improved significantly the quality of life of the patients (OSS and CMS scores), the Nottingham Surgilig was associated with improved radiographic survival and reduced rate of loss of post-operative radiographic CC distance. Accurate positioning of the coracoid endobutton seems to be among the most important factors preventing the loss of reduction.
